# Bacterial variation in the oral microbiota in multiple sclerosis patients

**DOI:** 10.1371/journal.pone.0260384

**Published:** 2021-11-30

**Authors:** Zahra Zangeneh, Ahya Abdi-Ali, Kianoosh Khamooshian, Amirhoushang Alvandi, Ramin Abiri

**Affiliations:** 1 Department of Microbiology, Faculty of Biological Sciences, Alzahra University, Tehran, Iran; 2 Research Center for Applied Microbiology and Microbial Biotechnology (CAMB), Alzahra University, Tehran, Iran; 3 Department of Neurology, School of Medicine, Kermanshah University of Medical Sciences, Kermanshah, Iran; 4 Department of Microbiology, School of Medicine, Kermanshah University of Medical Sciences, Kermanshah, Iran; 5 Medical Technology Research Center, Kermanshah University of Medical Sciences, Kermanshah, Iran; 6 Fertility and Infertility Research Center, Health, Health Technology Institute, Kermanshah University of Medical Sciences, Kermanshah, Iran; Mayo Clinic Rochester, UNITED STATES

## Abstract

**Background:**

Microorganisms in oral cavity are called oral microbiota, while microbiome consists of total genome content of microorganisms in a host. Interaction between host and microorganisms is important in nervous system development and nervous diseases such as Autism, Alzheimer, Parkinson and Multiple Sclerosis (MS). Bacterial infections, as an environmental factor in MS pathogenesis play role in T helper 17(Th17) increase and it enhancing the production of pro-inflammatory cytokines such as Interlukin-21(IL-21), IL-17 and IL -22. Oral microbiota consists diverse populations of cultivable and uncultivable bacterial species. Denaturing gradient gel electrophoresis (DGGE) is an acceptable method for identification of uncultivable bacteria. In this study, we compared the bacterial population diversity in the oral cavity between MS and healthy people.

**Methods:**

From October to March 2019, samples were taken at Kermanshah University of Medical Sciences’ MS patients center. A total of 30 samples were taken from MS patients and another 30 samples were taken from healthy people. Phenotypic tests were used to identify bacteria after pure cultures were obtained. DNA was extracted from 1 mL of saliva, and PCR products produced with primers were electrophoresed on polyacrylamide gels.

**Results:**

The genera *Staphylococcus*, *Actinomyces*, *Fusobacterium*, *Bacteroides*, *Porphyromonas*, *Prevotella*, *Veillonella*, *Propionibacterium* and uncultivable bacteria with accession number *MW880919-25*, *JQ477416*.*1*, *KF074888*.1 and several other un-culturable strains were significantly more abundant in the MS group while *Lactobacillus* and *Peptostreptococcus* were more prevalent in the normal healthy group according to logistic regression method.

**Conclusion:**

Oral micro-organisms may alleviate or exacerbate inflammatory condition which impact MS disease pathogenesis. It may be assumed that controlling oral infections may result in reduction of MS disease progression.

## Introduction

The term of "microbiome" was firstly invented by Lederberg for defining commensal, symbiotic inhabitant microorganism inside the body or on the skin of eukaryotic organisms [1]. Microbiome consists of total genome content of microorganisms in a host [2]. Of note, nearly 1–3 percentage of body weight is composed of microbiome and this amount increases with aging. Most bacteria in skin, intestine, and mouth are resident, while intestinal microbiome is the most complex one [3, 4]. There are many reports, showing great impacts of human microbiome on several physiological processes and immune modulations, production of vitamins and antimicrobial compounds, and inflammation [2]. For example, the interactions between host and microorganisms are important in the pathogenesis of some diseases, including Autism, Alzheimer, Parkinson, and Multiple Sclerosis [2, 5]. Microorganisms in oral cavity have different names such as oral microflora, oral microbiota or oral microbiome [1]. According to the favourable temperature for bacterial growth, oral cavity composed of about 800 different bacterial species as follows: *Veillonella atypica*, *Porphyromonas gingivalis*, *Selenomonas* spp., *Aggregatibacter actinomycetemcomitans*, *Prevotella intermedia*, *Capnocytophaga* spp. *Streptococcus faecalis*, and *Lactobacilli* [3]. Many non-culturable micro-organisms which are only identifiable by molecular methods like 16S rRNA DGGE and Next Generation Sequencing (NGS) are resident in the oral cavity [1, 3]. There are several documents reporting the roles of oral microbiota in both the pathogenesis and prevention of many non-infectious diseases such as dental, cardiovascular, and respiratory diseases and also diabetes [3]. It has been previously shown that gum inflammations can increase the risk of developing throat and mouth cancers. Moreover, bacterial infections cause inflammation and change many signaling pathways like Nuclear Factor Kappa Beta (NFκB) pathway, consequently leading to cytokine induction and release of free oxygen radicals that could cause or induce malignancies directly or indirectly [6].

MS is an autoimmune disease associated with chronic inflammation and demyelination of nerve cells [7]. In this regard, infiltration of auto-reactive T cells into central nervous system (CNS) is known as the probable mechanism of MS development [7, 8]. Both Th1 and Th17 pathways were also found to be involved in MS pathogenesis and axonal demyelination [8, 9]. Besides the immune mechanisms, several environmental factors like low level of vitamin D, latitude, smoking and obesity may also be involved in MS pathogenesis. Since the etiology (ies) of MS has not been elucidated yet, some nonspecific therapeutic drugs including Interferon-beta (IFN-β), glatiramer acetate, and teriflunomide and dimethyl fumarate with many side effects are currently applied for the treatment of this disease [10]. Bacterial infections and gut microbiota have recently been discovered as the causative environmental factors in MS and Experimental autoimmune encephalomyelitis (EAE) pathogenesis [2, 5, 10]. Moreover, it was indicated that some pathogenic or commensal bacteria can activate Th17 cells [11]. Accordingly, Th17 cells trigger IL-21, IL-17, and IL-22 productions [8]. On the other hand, some members of gut microbiota can activate regulatory T lymphocytes (T-reg) cells [12–14]. Additionally, T-reg can produce anti-inflammatory cytokines like IL-10 which leads to attenuation of inflammatory conditions [7, 11]. So gut microbiota can regulate balance of pro-inflammatory T cell (Th1/Th17) and anti-inflammatory T cell (T-reg) in MS patients. Any change in intestine microbiota, like increase of *Acinetobacter calcoaceticus* may cause imbalance of immune homeostasis and lead to progression of MS disease [15].

DGGE is an acceptable method for identifying uncultureable bacteria. In the method, amplified DNA fragments with same length and different sequences are separated based on the differences in electrical charges. The amplified fragments are loaded on a polyacrylamide gel containing a linear gradient of denaturants such as formamide and urea. Afterward, two stranded DNA molecules are separated at a certain concentration of denaturants based on the GC’s content and sequences. Finally, there is a band pattern with each band represents one unique molecular sequence related to a single species [16].

The role of the oral microbiome in various diseases has been previously highlighted in several studies. As a result, it appears that examining the effects of the oral microbiome on MS patients’ prevention, treatment, and outcome may reveal some sorts of relationship with this disease. In this study, the oral microbiota of MS patients was characterized and compared with that of healthy people using culture and DGGE methods. The results of this study could be important in discovering new prevention and/or treatment methods for MS.

## Materials and methods

### Patients and specimens

The study was conducted in MS center of Kermanshah University of Medical Sciences from October 2019 to March 2020. Of note, Kermanshah province includes several cities with different populations. Thirty positive MS patients in terms of the clinical and Magnetic resonance imaging (MRI) criteria were introduced by the neurologist to be included in this study. All the patients were in remission phase of disease during sampling. Moreover, 30 documented healthy matched by age, sex, Body mass index (BMI), smoking status, and other criteria were enrolled as the control group. This study was approved by the ethics committees with code 3010408 in Kermanshah University of Medical Sciences. Written consent was obtained from all the included participants before sampling.

For culture method, two consecutive swab samples were taken from the side of the mouth of each person, one of which was soaked in *Phosphate-buffered saline* (PBS) buffer for aerobic conditions and the other was placed in thioglycollate broth for anaerobic conditions. furthermore, 1 ml saliva was collected in a sterile falcon for DNA extraction and Molecular methods. All the samples were transferred to the laboratory regarding cold chain transfer standards.

Exclusion criteria included antibiotic use in the previous three months, probiotic use in the previous month, corticosteroids use in the previous two weeks, other autoimmune diseases, periodontitis and being pregnant. Inclusion criteria included confirmation of the disease by a specialist based on MRI, patient history and physical condition. Patients and healthy groups were evaluated for periodontitis and did no periodontitis symptoms.

### Isolation and identification of the isolates

Brucella blood agar supplemented with hemin and vitamin K was used for primary cultivation of the isolates in both aerobic and anaerobic conditions (0.2%O_2_, 80% N_2_ in Anoxomat system) at 37°C for 18h and 72h, respectively. In order to identify the anaerobic strains, standard biochemical tests like gram staining, oxidase, catalase, nitrate reduction, hemolysis on blood agar, aerotolerance, susceptibility to kanamycin, vancomycin, rifampin and penicillin, sugar fermentation, bile esculin agar were carried out. Similarly, for identification of aerobic isolates biochemical tests like gram staining, catalase, acid production from glucose, oxidase, hemolysis on blood agar, susceptibility to bacitracin, furazolidone, novobiocin and optochin, salt tolerance, bile esculin agar, L-pyrrolidonyl arylamidase (PYR) tests were used [17].

### DNA extraction and 16SrDNA PCR

One ml of saliva was dissolved in 4ml PBS and DNA was extracted based on instruction of DNA extraction kit (FavorGen) and was stored at -20°C. Since the PCR product were subsequently applied for DGGE, at the start of forward primer, a GC clamp with 40 bases was added. Primers sequences for 16S rDNA are shown in Table 1 (Table 1). The thermal cycling was as follow: a first denaturation step: 94°C in 1 min followed by 30 cycles of denaturation at 94°C for 45 seconds, annealing at 53°C in 30sec, extension at 72°C for 35 seconds and a round of 5 min at 72°C as the final extension process. The PCR mixture contained 2X master mix (YektaTajhizAzma), 10pmol/ μl of each primer and 2 μl (50ng) DNA template. Accuracy of PCR reaction was characterized by agarose gel electrophoresis. At the next step, PCR products were separated by DGGE Electrophoresis.

**Table 1:** Sequence of primers [18].

Primer	Sequence
I-341f	CCTACGGGIGGCIGCA
I-533r	TIACCGIIICTICTGGCAC
GC clamp	CGCCCGCCGCGCGCGGCGGGCGGGGCGGGGGCACGGGGGG

### DGGE analysis of PCR products

Electrophoresis of PCR products were carried out on polyacrylamide gels as described by Muyzer et al [16, 18]. PCR products were loaded on polyacrylamide gel 10 (wt/vol) in 1X TAE (1X TAE is 0.04 M Tris base, 0.02 M acetic acid, and 1.0 mM EDTA [pH 7.5]). The denaturing gradient included 20% to 70% denaturants (100% denaturants consisted of 7 M urea and 40% formamide). The gels with different concentration of denaturants were added using a delivery wheel. Electrophoresis was performed for 17h at 60V and 60°C.

### Sequencing

A sample of any distinct band was sequenced and aligned in standard databases in order to detect any band on gel. For this purpose, the desired bands were cut with sterile scalpel and were placed in 50 μl 1X TAE. This mixture was stored at 4°C for 24h and PCR product was extracted with gel extraction kit. The sequencing process were carried out in Pishgam Company.

### Statistical analyzes

In order to analysis of the difference of bacteria between the specimens, χ square and Logistic Regression test were used in SPSS version 16.

## Results

### Patients

All patients who came to the collection center were women and 19–42 years old when they got sick. It was found 19 cases of patients group are overweight by investigation BMI. BMI average was 26.89 and 24.3 in patients and healthy groups, respectively. BMI average in patients group was higher than healthy normal group significantly (p value <0.001) which shows that overweight is a risk factors for MS severity (Table 2).

**Table 2:** Characteristics of MS patients and healthy group.

Bacteria	Patients Cases	Controls	Significance (P value)
Age	39.03	38.9	NS^1^
**Sex**			
Female	30	30	NS
Male	0	0	NS
**BMI (kg/m** ^**2**^ **)**	26.89	24.3	≤(0.001)*
**Smoking**	0	0	NS

* Significant, 1: Not Significant.

### Bacterial isolates

Bacteria Isolated from saliva and swab samples were studied by phenotypic tests. As a result, the genera *Staphylococcus*, *Streptococcus*, *Enterococcus*, *Micrococcus*, *Peptostreptococcus*, *Actinomyces*, *Lactobacillus*, *Fusobacterium*, *Bacteroides*, *Porphyromonas*, *Prevotella*, *Veillonella*, *Propionibacterium*, and *Bifidobacterium* were eventually detected. The number of all the above-mentioned bacteria were more in the patient group (p value <0.001), except *Peptostreptococcus* and *Lactobacillus*. *Micrococcus* and *Enterococcus* were higher in the healthy group, but they were not significant. *Prevotella* and *Propionibacterium* genera were only found in patients’ samples using cultural methods and *Streptococcus* genus was found in all cases.

### PCR-DGGE and sequencing

**Fig 1:**
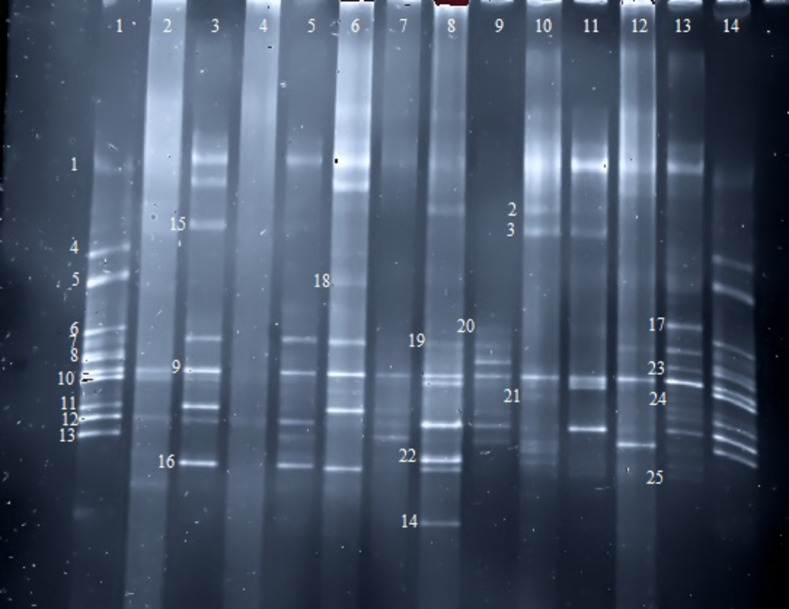
DGGE of PCR product; any band shows any bacterial genus or species. This method identified a greater number of bacteria than the cultural method. Sequencing detected B5, B6, S5, S6 etc. bands in DGGE method (not culture). Bacteria numbers: 1(Bacteroides), 2(B5), 3(B6), 4(Bifidobacterium), 5(Staphylococcus), 6(Peptostreptococcus), 7(Actinomyces), 8(Lactobacillus), 9(Streptococcus), 10(Micrococcus), 11(Prevotella), 12(Lactobacillus), 13(B10), 14(Porphyromonas), 15(S5), 16(S6), 17(S8), 18(B7), 19(B8), 20(S10), 21(S9), 22(B10), 23(B11), 24(S12), 25(B12), 26(Enterococcus). wells 1 and 14 were mix of bacteria. 3,5,7,9,11,13 were patient samples. 2,4,6,8,10,12 were healthy control.

DGGE findings showed that this method can detect a greater number of bacterial genera or species compared to cultural methods, as evidenced by the fact that at least 10 different bacteria are isolated in each sample, whereas the number of bacteria isolated using culture methods is significantly lower (Fig 1). It is noteworthy that the highest number of bacteria found in a person was 20 isolates using Relapsing Remitting Multiple sclerosis (RRMS) since 2014 and the lowest one was 3 isolates that belonged to a healthy person with no underlying diseases. Based on the total data obtained using culture and DGGE methods, *Lactobacillus* and *Peptostreptococcus* were significantly higher in the healthy group with Odds ratio equal to 0.039, 0.250 respectively compared to the patient group, *Micrococcus* and *Enterococcus* were more in healthy group but were not significant while the other genera were significantly higher in the patient group (p value <0.001) except *Bifidobacterium* that was higher in patient group but this difference wasn’t significant. Furthermore, *Veillonella* genus was only found in the patient group (Table 3). In general, the number of the detected bacteria were significantly greater in the patient group.

**Table 3:** Bacterial genera in patient and healthy groups.

Bacteria	Patients Cases	Controls	Significance (P value)	Odd Ratio
	N (%) genera	N (%) genera		
*Staphylococcus*	25 (%10.5)	9(%5)	≤(0.001)*	11.6
*Micrococcus*	10 (%4.2)	16 (%8.8)	NS^1^	0.438
*Streptococcus*	30 (%12.6)	30 (%16.5)	NS	1
*Enterococcus*	10 (%4.2)	15 (%8.2)	NS	0.5
*Lactobacillus*	16 (%6.9)	29 (%16.02)	≤(0.001)*	0.039
*Fusobacterium*	15 (%6.7)	2 (%1.1)	≤(0.001)*	9
*Porphyromonas*	16 (%6.9)	6 (%3.3)	≤(0.001)*	4.57
*Bacteroides*	29 (%11.8)	18 (%9.9)	≤(0.001)*	19.3
*Prevotella*	28 (%12)	16 (%8.8)	≤(0.001)*	12.25
*Veillonella*	4 (%1.7)	0 (%0)	≤(0.001)*	2.48
*Actinomyces*	23 (%9.7)	14 (%7.7)	≤(0.001)*	3.75
*Propionibacterium*	10 (%4.2)	3 (%1.6)	≤(0.001)*	4.5
*Bifidobacterium*	15 (%6.3)	8 (%4.4)	NS	2.7
*Peptostreptococcus*	6 (%2.5)	15 (%8.2)	≤(0.001)*	0.250

* Significant, 1: Not Significant.

### Sequencing

Some of bands on polyacrylamide gels were cut and sequenced. By sequencing it was found that some of them belong to uncultivable bacterial species indicating that the oral microbiome is diverse. These bands were 48 cases that after remove low quality reads 36 cases were aligned and 7 cases were submitted in https://www.ncbi.nlm.nih.gov/nuccore/ MW880919-25. These data with accession number were shown in Table 4. Among uncultivable bacteria B1, B4, B6, S20 and B16 in patient group and B13, S15, B20, S9, S17 and S25 in healthy group were higher significantly but other detected bacteria differences were not significant (p value >0.01) (Table 4).

**Table 4:** Bacterial species isolated from polyacrylamide gel based on DGGE method.

Bacteria	Patients Cases	Controls	Significance (P value)	Accession number or ID submission	Odd Ratio
	N (%) genera	N (%) genera			
B1	8 (%6.01)	1 (%0.7)	≤(0.001)*	MW880919 (submitted)	8.8
B2	4 (%3)	4 (%2.8)	NS^1^	JQ477416.1	1.3
B4	9 (%6.7)	2 (%1.4)	≤(0.001)*	KF074888.1	12.4
B5	5 (%3.75)	3 (%2.1)	NS	MW880920 (submitted)	1.3
B6,S20	11 (%8.3)	3 (%2.1)	≤(0.001)*	JX069809.1	4.5,0.0
B7	3 (%2.25)	3 (%2.1)	NS	MW880921 (submitted)	2.07
B8	2 (%1.5)	3 (%2.1)	NS	JQ449863.1	1
B9	4 (%3)	3 (%2.1)	NS	KF599923.1	1
B10	4 (%3)	3 (%2.1)	NS	GQ089813.1	1.5
B11	3 (%2.25)	3 (%2.1)	NS	JF178594.1	0.64
B12	2 (%1.5)	2 (%1.4)	NS	JQ460583.1	1
B13,S15	2 (%1.5)	10 (%7.04)	≤(0.001)*	EF508938.1	0.167,0.48
B16	10 (%7.5)	1 (%0.7)	≤(0.001)*	JF198912.1	12.4
B17,S5,S6,S10	9 (%6.76)	8 (%5.6)	NS	MT613456.1	3.2,1.5,0.64,0.48
B18	3 (%2.25)	3 (%2.1)	NS	MW880922 (submitted)	1
B19	5 (%3.75)	5 (%3.5)	NS	JF052766.1	1
B20	2 (%1.5)	11 (%7.7)	≤(0.001)*	CP054883.1	0.143
B21,S4	4 (%3)	5 (%3.5)	NS	JQ460619.1	0.64,1
B22	3 (%2.25)	2 (%1.4)	NS	HM265276.1	2.07
B23	6 (%4.5)	3 (%2.1)	NS	JF195658.1	1.62
S1	4 (%3)	4 (%2.8)	NS	MW880923 (submitted)	4.4
S2	4 (%3)	4 (%2.8)	NS	MN400008.1	1
S3	5 (%3.75)	4 (%2.8)	NS	KY436503.1	1.30
S7	1 (%0.75)	4 (%2.8)	NS	MT138558.1	0.3
S8	1 (%0.75)	1 (%0.7)	NS	KY623059.1	1
S9	2 (%1.5)	10 (%7.04)	≤(0.001)*	JQ462822.1	0.167
S11	2 (%1.5)	2 (%1.4)	NS	MW880924 (submitted)	1
S12	3 (%2.25)	4 (%2.8)	NS	MW880925 (submitted)	0.7
S14	3 (%2.25)	1 (%0.7)	NS	KF101581.1	1
S16	1 (%0.75)	2 (%1.4)	NS	KF067341.1	1
S17	2 (%1.5)	8 (%5.6)	≤(0.001)*	HM269358.1	0.196
S19	1 (%0.75)	2 (%1.4)	NS	KC708270.1	0.48
S21	2 (%1.5)	2 (%1.4)	NS	EU986018.1	1
S22	2 (%1.5)	4 (%2.8)	NS	LC358461.1	0.6
S23	0 (%0)	2 (%1.4)	NS	JQ818398.1	0
S25	1 (%0.75)	10 (%7.04)	≤(0.001)*	MT597603.1	.080

* Significant, 1: Not Significant

## Discussion

Host-microbiome interactions play important roles in both progression and regression of several autoimmune diseases such as Inflammatory Bowel Disease, Rheumatoid Arthritis, Multiple Sclerosis, and EAE. Although the main causes of MS are not elucidated yet, the roles of genetic and environmental factors were confirmed earlier. In this regard, one may point toward several environmental factors such as resident microbiota, overweight and obesity, smoking, and low level of vitamin D. Inflammation inducing factors in CNS like microbiota, are also considered as major contributors to establish the disease [7]. Most of the studies concerning the interactions between microbiome and MS are focused and limited to gut microbiome [5, 8, 11, 12]. The composition of gut microbiota is different between healthy and patient groups even in similar environmental conditions, which shows an increase in *Firmicutes*, *Bacteroidetes* and *Actinobacteria* and a decrease of *Proteobacteria* [19]. *Akkermansia* as one of the gut microbiota members is inversely related to patients’ EDSS and MRI and it may have a beneficial effect on MS. Administration of MS-derived *Akkermansia* to mice reduced MS induction rate [20]. As the majority of oral bacteria are uncultivable, the uses of molecular techniques such as DGGE or NGS are obligatory to identify the members of the microbiota. This study aimed to determine and compare the oral microbiota between MS patients and healthy individuals. In this regard, the saliva samples obtained from the two study groups were collected and cultured. In addition, the extracted total DNA molecules were subjected to PCR-DGGE for further identifications.

All the included patients were overweight 20–40 year old females. The members of the *Staphylococcus*, *Fusobacterium*, *Bacteroides*, *Porphyromonas*, *Prevotella*, *Veillonella*, *Actinomyces*, *Propionibacterium*, and *Bifidobacterium* genera were observed to be higher in the patient group, while *Peptostreptococcus* and *Lactobacillus* were significantly higher in the control group. Inflammation caused by bacteria may change many signaling pathways such as NF-κB, Mitogen-activated protein kinases (MAPK), Th1, and Th17 pathways, which were reported to be associated with cytokine induction [6]. Production of IL-1, IL-6, IL-21, IL-17, and IL-22 enhances inflammatory condition among MS patients, while regulatory T-cell releasing cytokines like IL-10 may alleviate this condition [7, 8, 11]. On the other hand, fiber consumption by some bacteria can induce a number of immune system regulatory metabolites like short-chain fatty acid (SCFA), inducing colonic regulatory T-cells [12].

Based on our results in this study, *Lactobacillus* and *Peptostreptococcus* were observed to be significantly higher in the saliva specimens obtained from the healthy group compared to the MS patients with odd ratios equal to 0.039, 0.250 respectively. Correspondingly, this may show their positive effects on the inhibition of MS pathogenesis. *Lactobacili* were less prevalent in different specimens obtained from the patients suffering from Oral Cancer, gingival inflammation, and Pancreatic Cancer [21–24]. *Lactobacillus* inhibited Oral Cancer by reducing the expression of MAPK signaling pathway involved in the initiation of cancer [25]. *L*. *salivarius* could decrease the reduction process of carcinogenic 4-nitroquioline 1-oxide (4NQO), thereby reducing DNA oxidative damage [26]. *Lactobacillus* is significantly higher in oral cavity of normal healthy people comparing to MS patients group (Odds Ratio: 0.039). *Enterococcus* is known as a probiotic bacterium that can reduce the severity of MS diseases by the stimulation of T cell differentiation to T-reg cells [27] although *Enterococcus* was higher in healthy group but not significant. *Peptostreptococcus* produces indoleacrylic acid (IA) by metabolizing tryptophan and this metabolite consequently increases the IL-10 secretion [28]. On the other hand, *Prevotella*, *Porphyromonas*, *Bacteroides*, *Actinomyces*, *Staphylococcus*, *Fusobacterium Veillonella*, and *Propionibacterium* genera were found to be significantly more abundant in the patient group (odd ratios 12.25, 4.57, 19.3, 3.75, 11.6, 9, 2.48, 4.5 respectively). *Bifidobacterium* was more in patient group but it wasn’t significant. Many species of *Bifidobacterium* genus are used for probiotic therapy, especially intestine living species; however, their effects can vary among different species [29]. Although *Bifidobacterium* can induce T-reg cells [30], it may not have a beneficial effect on MS patients who do not possess sufficient T-reg cells [31]. According to the report by Toghi et al., *Bifidobacterium* mostly appears in oral cavity of newborns and plays an important role in the maturation of immune system, so it might be involved in the MS pathogenesis [32]. Even in the absence of extracellular carbohydrates, *Bifidobacterium* produces acid in oral cavity. Thereafter, the produced acid leads to dental caries and inflammatory responses, which are known as the promoters of MS pathogenesis. Additionally, the main acidic metabolite of *Bifidobacterium* is acetic acid, which has a higher pKa compared to lactic acid produced by *Lactobacillus* and *Streptococcus* as well as better penetration and decoying effects on dental enamel [33, 34]. Notably, *Prevotella copri* is more abundant in the intestine of patients with Inflammatory Bowel Disease and Rheumatoid Arthritis. *Prevotella*, together with higher fiber consumption diet, leads to higher production of butyrate, which in turn ameliorates EAE induction in EAE mouse model. Furthermore, butyrate activates aryl-hydrocarbon receptor that reduces IL-17 production by Th17 [35]. Two studies reported the reduced frequency of *Prevotella copri* and *Prevotella histicola* in the MS patients’ intestine that can attenuate MS severity [13, 14]. The simultaneous administration of *Prevotella histicola with* Copaxone (Glatiramer acetate-GA) attenuated the diseases’ induction in mice [36]. These finding showed the role of *Prevotella* in intestine; however, in oral cavity, *Prevotella* species such as *Prevotella Nigrescens* and *Prevotella intermedia* can activate inflammatory response through Toll-like receptor 2 as well as inducing epithelial cells to produce inflammatory cytokines [37, 38]. Oral microenvironment may turn to acidic pH in some diseases like gout, which is a favorable property for the growth of *Prevotella intermedia* followed by the stimulation of inflammatory pathways [39]. The lipid A structure of *Porphyromonas* Lipopolysaccharides (LPS) interferes with immune system and consequently induces several diseases [38]. This structure activates NF-κB signaling pathway, leading to the inflammatory cytokines’ secretion [40]. The data obtained from the animal models showed the role of *Porphyromonas* in the pathogenesis of systemic inflammation, Alzheimer disease, different types of Cancers, and Atherosclerosis [41–43]. In Alzheimer disease, oral pathogens like *Porphyromonas gingivalis*, grows under inflammatory conditions and with inflammatory cytokines’ production, it may affect glial cell activation [43]. Some of intestinal *Bacteroides* and *Lactobacillus* species can reduce inflammation by producing SCFA, but studying saliva samples confirmed the role of *Bacteroides* in Pancreas Cancer [24]. *Bacteroides fragilis* has also been implicated in the pathogenesis of some other diseases such as Autism and Alzheimer diseases, with the pathogenesis linked to the pro-inflammatory effects of LPS, capsule, agglutinin, and fimbriae [35, 44–46]. Staphylococcal super-antigens trigger the CD4 T-cell activation, increase auto-reactive T-cells, and leads to the exacerbation of autoimmune disease. In addition, the correlation between staphylococcal toxins and the pathogenesis of MS were confirmed by an EAE model. As well, super-antigens are involved in the immune-mediated diseases, Rheumatoid Arthritis, Psoriasis, and Diabetes [47–50]. *Fusobacterium* has been previously linked to Colon Cancer and Ulcerative Colitis [5]. Bacterial LPS is the most common cause activating inflammatory signaling pathways, apparently as seen in *Fusobacterium*, *Veillonella*, and *Propionibacterium*, in which LPS induces inflammatory conditions through Toll-like receptors (TLRs) and NFκB pathways leads to the IL-1, IL-6, IL-8, and IL-17 production [51, 52]. Moreover, this can be associated with pain and sensitivity in the oral cavity; however, *Fusobacterium* gavage has been shown to reduce the *Lactobacillus* level in MS rat’s model [53]. In concordance with our results, Boullerne and colleagues detected the *Porphyromonas*, *Fusobacterium*, *Actinomyces*, *Prevotella*, *Veillonella* and *Streptococcus* genera in oral cavity of a twin MS patient [54].

## Conclusion

Oral microorganism’s interaction may exacerbate inflammatory condition. In MS patients *Staphylococcus*, *Fusobacterium*, *Bacteroides*, *Porphyromonas*, *Prevotella*, *Veillonella*, *Actinomyces*, *Propionibacterium* and *Bifidobacterium* genera were higher and *Peptostreptococcus*, *Micrococcus*, *Enterococcus* and *Lactobacillus* were lower. On the other hands, Logistic Regression confirms relation between these bacteria with a higher probability of developing MS. Finally, production of pro-inflammatory cytokines such as Th17, IL-21, IL-17 and IL-22, is the main mechanism MS disease progression through activating T-cell by bacteria.

## Supporting information

S1 Raw image Uncropped and unadjusted of gel results. (PDF)
