# Supplementary figures and images for: Bacterial variation in the oral microbiota in multiple sclerosis patients
Source: PLoS One. 2021 Nov 30;16(11):e0260384. doi: 10.1371/journal.pone.0260384 (PMC8631616; doi:10.1371/journal.pone.0260384)

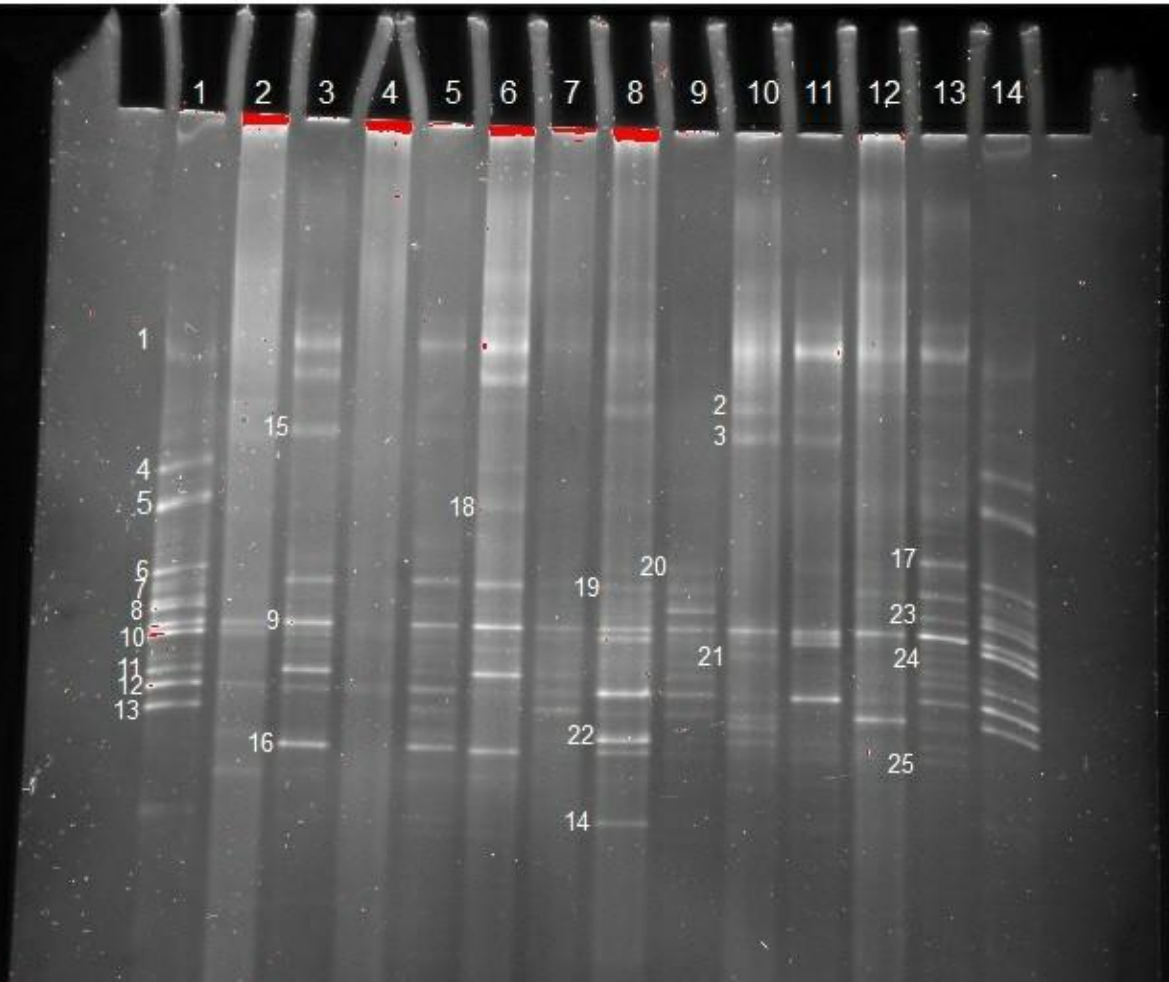

Supplement: S1 Raw image — (PDF) [file pone.0260384.s001.pdf]
